# Heart-Shaped lung hydatid cyst in a chest radiograph

**DOI:** 10.1590/0037-8682-0099-2022

**Published:** 2022-06-06

**Authors:** Yener Aydin, Hayri Ogul, Atilla Eroglu

**Affiliations:** 1 Department of Thoracic Surgery, Ataturk University, Medical Faculty, Erzurum, Turkey.; 2 Department of Radiology, Duzce University, Medical Faculty, Duzce, Turkey.

A 36-year-old woman presented for evaluation of chest pain. Posteroanterior direct radiography showed a homogeneous opacity in the middle zone of the left lung. Lateral chest radiography showed a heart-shaped opacity with smooth edges in the posterior of the left lung (Figure 1). Given these findings, the patient underwent a left thoracotomy. In addition, cystotomy and capitonnage were performed on the hydatid cyst in the superior segment of the left lower lobe.

**FIGURE 1:**
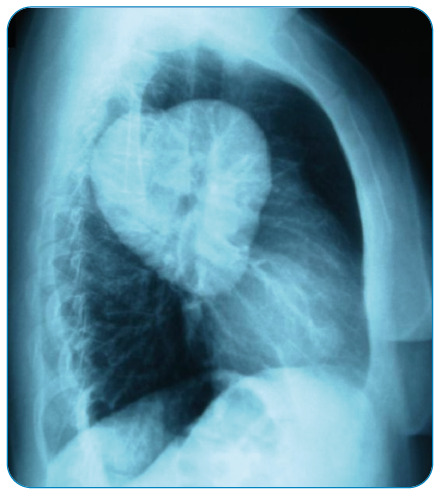
Lateral direct radiograph shows a hydatid cyst resembling a heart.

Hydatid cyst disease is an important parasitic disease seen in areas where agriculture and animal husbandry are carried out. The most common pulmonary clinical manifestations are cough, chest pain, dyspnea, and hemoptysis^1^ ^,^ ^2^. Clinical findings and radiological examinations are usually sufficient for diagnosis in areas where the disease is endemic. Hydatid cysts may present with different radiological appearances depending on the affected organ and the site, nature, and size of the cyst. Treatment is surgical, and the use of albendazole is recommended to prevent postoperative recurrences^3^.

## References

[1] Aydin Y, Ulas AB, Ince I, Korkut E, Ogul H, Eren S (2021). Large Case Series Analysis of Cystic Echinococcosis. Indian J Surg.

[2] Aydin Y, Altuntas B, Kaya A, Ulas AB, Uyanık MH, Eroglu A (2018). The Availability of Echinococcus IgG ELISA for Diagnosing Pulmonary Hydatid Cysts. Eurasian J Med.

[3] Aydin Y, Ulas AB, Ince I, Kalin A, Can FK, Gundogdu B (2022). Evaluation of albendazole efficiency and complications in patients with pulmonary hydatid cyst. Interact Cardiovasc Thorac Surg.

